# Constructing Integrated Networks for Identifying New Secondary Metabolic Pathway Regulators in Grapevine: Recent Applications and Future Opportunities

**DOI:** 10.3389/fpls.2017.00505

**Published:** 2017-04-12

**Authors:** Darren C. J. Wong, José Tomás Matus

**Affiliations:** ^1^Ecology and Evolution, Research School of Biology, Australian National UniversityActon, ACT, Australia; ^2^Centre for Research in Agricultural Genomics, CSIC-IRTA-UAB-UBBarcelona, Spain

**Keywords:** *Vitis vinifera*, stilbenes, flavonoids, ripening, systems biology, genome-wide, multi-omics, data integration

## Abstract

Representing large biological data as networks is becoming increasingly adopted for predicting gene function while elucidating the multifaceted organization of life processes. In grapevine (*Vitis vinifera* L.), network analyses have been mostly adopted to contribute to the understanding of the regulatory mechanisms that control berry composition. Whereas, some studies have used gene co-expression networks to find common pathways and putative targets for transcription factors related to development and metabolism, others have defined networks of primary and secondary metabolites for characterizing the main metabolic differences between cultivars throughout fruit ripening. Lately, proteomic-related networks and those integrating genome-wide analyses of promoter regulatory elements have also been generated. The integration of all these data in multilayered networks allows building complex maps of molecular regulation and interaction. This perspective article describes the currently available network data and related resources for grapevine. With the aim of illustrating data integration approaches into network construction and analysis in grapevine, we searched for berry-specific regulators of the phenylpropanoid pathway. We generated a composite network consisting of overlaying maps of co-expression between structural and transcription factor genes, integrated with the presence of promoter *cis*-binding elements, microRNAs, and long non-coding RNAs (lncRNA). This approach revealed new uncharacterized transcription factors together with several microRNAs potentially regulating different steps of the phenylpropanoid pathway, and one particular lncRNA compromising the expression of nine stilbene synthase *(STS)* genes located in chromosome 10. Application of network-based approaches into multi-omics data will continue providing supplementary resources to address important questions regarding grapevine fruit quality and composition.

## Network-based approaches into omics data

Complex biological processes can be studied from a “multi-omics” perspective thanks to the recent improvements in genome-wide techniques and systems biology approaches. Each omics data type is particularly useful in elucidating the constituents and function of a particular cellular domain. Together, they constitute layers of biological complexity. Genomic data generated from genome sequencing projects are commonly used to ascribe molecular function and biological processes based on sequence similarity, while transcriptomics and metabolomics data typically provide a global “snapshot” of gene expression and metabolite dynamics in various biological contexts.

For many omics data, interactions/associations between molecules can be represented as networks, where nodes (genes, proteins, metabolites) are connected by edges. These edges denote an association often inferred from correlational and informational theoretic measures such as Pearson correlation coefficient (PCC) and mutual information (MI), respectively. In the case of gene co-expression networks (GCNs), edges represent similar gene expression behaviors. Based on the “guilt by association” principle, genes involved in related processes share similar gene expression dynamics across a wide range of experiments (Wolfe et al., 2005). However, as functional information could be delimited to a reduced number of interactions within a gene network (Gillis and Pavlidis, 2012), subsequent targeted gene characterizations are needed to prove these relationships. Whether the function of a network is dependent or not on specific interactions, GCN analysis have proven to be a powerful tool for inferring gene function and coordinated biological processes related to plant metabolism (Persson et al., 2005; Itkin et al., 2013).

Other forms of networks constructed from omics datasets do not necessarily rely on abundance or expression levels to establish node relationships. For example, protein-protein interaction networks describe physically interacting protein pairs identified from high-throughput yeast two-hybrid screens (e.g., Arabidopsis Interactome Mapping Consortium, 2011). Also, genome-wide location studies (i.e., by using ChIP-Seq) allow determining regulatory networks for transcription factors (TF) and other DNA-binding proteins. These TF-binding networks have led to the identification of novel components and of new connections that alter the network diagrams originally drawn by genetic and molecular analyses (reviewed by Ferrier et al., 2011).

## Recent application of metabolite network-based approaches in grapes

Studies utilizing networks constructed from omics data profiled in the berry are continuously increasing (Table 1). Network analyses involving metabolite datasets (primary and/or secondary metabolites) are by far the most reported. These studies have included networks inferred from single contexts such as berry development and ripening (Zamboni et al., 2010; Dai et al., 2013; Wang et al., in press), or in combination with other factors including environmental influence (Guan et al., 2016; Savoi et al., 2016; Reshef et al., 2017), and/or cultivar differences (Degu et al., 2014; Cuadros-Inostroza et al., 2016). Network topology has also been investigated in detail to reveal critical metabolites and their regulation. For instance, Cuadros-Inostroza et al. (2016) showed that an increase in network connectedness and density (especially regarding primary metabolites) became prevalent at specific berry developmental stages such as fruit set and veraison (i.e., the onset of ripening). The same study, in concordance with Degu et al. (2014), highlighted that berry-metabolite networks from different cultivars could possess contrasting network topologies, albeit with overall network connections generally maintained. Metabolite networks from the cultivars cv. “Merlot” (Cuadros-Inostroza et al., 2016) and cv. “Shiraz” (Degu et al., 2014) were consistently denser to that off cv. “Cabernet Sauvignon.”

**Table 1:** **Studies of grape berry development and composition involving molecular networks approaches**.

**Network type**	**References**	**Network approach and/or main findings**
**METABOLITE**		
M	Dai et al., 2013	Identification of primary metabolic switches during berry development to suggest timing of regulation in carbohydrate metabolism. Partial correlation network identified novel connections between metabolites (e.g., trehalose-6-phosphate and succinate).
M	Degu et al., 2014	Identification of berry transcript/metabolite differences between two black-skinned cultivars in relation to the phenylpropanoid pathway and stress-related hormones. Metabolic network analysis (based on correlation matrices) suggested a tighter metabolic control in cv. “Shiraz” compared to cv. “Cabernet Sauvignon.”
M	Cuadros-Inostroza et al., 2016	Use of untargeted metabolic profiling to identify the main primary metabolite changes during grape berry development in two black-skinned cultivars. Network connectivity of primary metabolites showed a stage- and cultivar-dependent accumulation pattern (e.g., opposite behavior between cultivars in sugar and amino acids), suggesting differences in primary metabolism regulation.
M	Reshef et al., 2017	Pearson correlation-based networks revealed that light and temperature perturbations modified primary and secondary metabolic composition between berries of a single cluster, increasing the number of negative correlations between metabolites in both pulp and skin.
**GENE**		
G + R	Wong et al., 2013	Development of a microarray-derived online platform (VTCdb; http://vtcdb.adelaide.edu.au/Home.aspx) for transcriptional regulatory inference, offering a ranked list of co-expressed genes, functional annotations and Gene Ontology enrichment analyses. Condition dependent (berry/stress) and independent analyses can be used to find co-expression modules.
G	Wang et al., 2015	Targeted GCN based on *a priori* information in Arabidopsis. Gene co-expression analysis between WRKY and VQ gene families using the cv. “Corvina” atlas showed clear sub-networks related to postharvest withered berries.
G	Wen et al., 2015	Gene co-expression networks derived from RNA-Seq data were employed to identify transcription factors that could regulate terpene synthesis.
G + R	Moretto et al., 2016	Development of the VESPUCCI gene expression compendium (http://vespucci.colombos.fmach.it) as an exploratory tool for modular gene expression responses based on sample contrast matrices. Microarray and RNA-Seq expression data from grape is integrated, largely representing berry development studies.
**OTHER**		
ta-siRNA	Zhang et al., 2012	Using a compendium of grape small RNA and degradome libraries, conserved and grapevine-specific trans-acting small interfering RNAs (ta-siRNA) were identified. Several ta-siRNA regulatory cascades were associated to metabolism, stress response, and development processes.
miRNA + R	Belli Kullan et al., 2015	Deep characterization of microRNA across a large compendia of grape tissues and developmental stages. Many novel and cultivar-specific miRNAs were also defined. The combination of miRNA expression dynamics and target inference reinforced their role in the regulation of berry development and hormonal regulatory circuitry. An online platform was created for further exploration (grape sRNA atlas DB; https://mpss.danforthcenter.org/dbs/index.php?SITE= grape_sRNA_atlas).
miRNA + R	Pulvirenti et al., 2015	Design of BIOWINE (http://alpha.dmi.unict.it/biowine/) as a genome/RNA-Seq data tool that exploits third-party databases to perform gene set enrichment analysis. There is special emphasis in the functional analysis of grapevine genomes of cultivars under environmental stress in connection with microRNA data.
**INTEGRATED**		
I (G, M, Prot)	Zamboni et al., 2010	Multi-omics data from four berry developmental stages and three postharvest withering intervals of cv. “Corvina” were integrated by hierarchical clustering to identify stage-specific metabolites, transcripts and proteins as putative biomarkers.
I (G, miRNA)	Palumbo et al., 2014	Use of network-based methods to analyze large-scale gene expression data in tomato and grape led to the identification of switch genes within “fight-club” nodes that may act as master regulators in berry developmental/ripening transitions.
I (G, M)	Savoi et al., 2016	Network analyses derived from RNA-Seq and targeted phenylpropanoid/isoprenoid metabolite data showed a tight transcriptional regulatory mechanism controlling berry monoterpene production under prolonged drought.
I (G, M)	Guan et al., 2016	Targeted quantification of metabolites, hormones and transcripts were assembled in correlation networks to study the effect of light exclusion on berry skin and pulp composition in red-skinned and red-fleshed cultivars.
I (G, Prom)	Wong et al., 2016	Identification of the TOP100 co-expressed genes and most representative biological functions of the complete R2R3-MYB family. Screen of MYB binding motifs in the promoters of all co-expressed genes. Identification of a new regulator of stilbene accumulation by using composite networks.
I (G, Prom)	Loyola et al., 2016	Combined analysis of microarray and RNA-Seq data with promoter inspections to identify HY5 and HYH community gene co-expression and *cis*-regulatory sub-networks. Search of potential gene targets identified a preferential regulation of the flavonol biosynthetic pathway and processes related to DNA/protein repair, jasmonic acid, photosynthesis and terpenes.
I (M, Prot)	Wang et al., in press	Analysis of metabolite and protein dynamics over a large developmental time course by using Granger causality associations revealed the occurrence of time-shift correlations within and between metabolite and protein networks.
I (G, Prom)	Wong et al., 2017	Identification of *cis*-regulatory element (CRE)-driven modules in condition-specific (stress) gene co-expression networks, generated from microarray and RNA-seq data.

*G, genes; M, metabolite; Prot, protein; Prom, promoter; + R, includes resource/database; I, integrated (G, M, Prot, Prom, miRNA inclusive)*.

Rewiring of berry metabolite networks under different environmental conditions or perturbations such as drought (Savoi et al., 2016) and sunlight exposure (Reshef et al., 2017) have also been reported. These studies have shown that higher network connectivity is commonly observed in perturbed networks. Such property could be associated to a tighter metabolic control of the metabolic pathways under investigation. Such is seen for phenylpropanoid and volatile organic compounds (VOC) in berries under prolonged drought compared to non-stress berries (Savoi et al., 2016). Similarly, primary metabolite networks encompassing compounds related to glycolysis, the TCA cycle, and amino acid metabolism showed higher network connectivity in shaded berries compared to fully exposed berries (Reshef et al., 2017).

Some metabolic-network studies have shown that certain metabolites (or classes) could act as important switches in the developmental regulation of metabolism during berry growth and ripening, given their high centrality (number of connections) or degree scores in their network. Dai et al. (2013) showed that trehalose-6-phosphate appeared to be the most connected compound in the primary metabolite network of cv. “Cabernet Sauvignon” grapes, with significant partial correlations to sugar metabolism, glycolysis, and TCA cycle intermediates. Altogether, these compounds may be implicated in coordinating metabolite dynamics during berry development. One recent study highlighted fucose as critical for coordinating metabolic regulation in a stage-specific manner, thus deprioritizing the importance of sugars such as glucose, fructose, and sucrose as a function of network centrality measure (Cuadros-Inostroza et al., 2016). These findings further demonstrate the complexity of berry metabolic regulation during development and ripening.

## Gene co-expression networks to study grape berry ripening

The increased ease of transcriptome profiling, combined with availability of datasets shared by the grapevine research community in public repositories, has led to increased attention in the use of gene co-expression networks (GCNs) in the study of berry development and metabolism. GCNs can be classified into “condition-dependent” and “condition-independent” categories (Usadel et al., 2009). In grapes, several studies have focused on condition-independent GCNs (encompassing different cultivars, tissues, developmental stages, stress and vineyard management treatments) as it provides a more convenient and representative (albeit “static”) relationship overview (Table 1). This approach has been useful for ascribing the most representative biological functions of the 134 grapevine R2R3-MYB transcription factors based on their top 100 co-expressed genes (Wong et al., 2016), where VviMYB13 (close homolog of VviMYB14 and VviMYB15) was identified as an additional *STILBENE SYNTHASE* regulator acting in a tissue- and/or stress-dynamic manner.

Platforms such as the ViTis Co-expression DataBase (VTCdb; Wong et al., 2013) and VESPUCCI (Moretto et al., 2016) have been successfully exploited to study the extent of transcription factor regulatory networks, providing support for targeted functional studies. Such is the case for the bZIP TF VvibZIPC22, which is involved in the regulation of flavonoid biosynthesis in grapes and may be also implicated in carbohydrate and amino acid metabolism, as inferred from VESPUCCI (Malacarne et al., 2016). Two other bZIP TFs (VviHY5 and VviHYH) were shown to co-operatively mediate flavonol accumulation in grapes in response to sunlight and ultraviolet radiation exposure (Loyola et al., 2016). As inferred from VTCdb and GCN analysis, these regulators were potentially implicated in carbohydrate and isoprenoid metabolism in addition to the control of the flavonoid pathway. Similarly, the involvement of the grapevine VviWRKY26 in the regulation of vacuolar transport and flavonoid biosynthesis was demonstrated using a combination of transcriptomic approaches including GCNs (Amato et al., 2017).

Condition-dependent GCNs have been constructed from tissue- or stress-specific datasets, including berry (Zamboni et al., 2010; Palumbo et al., 2014) or abiotic and biotic stresses (Wong et al., 2017). These GCNs provide several advantages over condition-independent networks as inferring gene function is largely simplified, providing a more “dynamic” overview of gene relationships that otherwise could be enhanced or lost in certain conditions (Obayashi et al., 2011). One example of a condition-specific GCN involves the study of the transcriptomes of five black-skinned cultivars across four berry phenological stages (Palumbo et al., 2014). The authors identified “fight-club” nodes and “switch” genes, having the latter unique expression profiles and network topological properties, such as a marked negative correlation connectivity to both neighboring genes and genes grouped to other modules in the network. Genes associated with transcription factor activity; cell wall modification and carbohydrate and secondary metabolism were found as candidate master regulators, potentially inducing large-scale transcriptome reprogramming during berry development (Palumbo et al., 2014).

Finally, miRNA and siRNA-mediated gene regulatory networks have also been constructed from high-throughput small RNA and degradome sequencing and computational target prediction methods (Zhang et al., 2012; Belli Kullan et al., 2015). These networks (not relying in abundance or expression levels) revealed novel modules such as miR156/miR172 regulatory circuits and VviTAS3/4 regulatory cascades, which are implicated in regulating plant growth and development and in the control of flavonoid biosynthesis, respectively.

## Toward the integration of multi-omics data in grapes

Although individual omic network methods have been widely used, a shift toward multi-omics data and integration is increasingly being adopted in plant biology (Proost and Mutwil, 2016), including grapevine (Table 1). Integration approaches allow building complex maps of molecular regulation and interaction. By these means, complex traits from these networks can be assessed (e.g., plasticity and evolution).

The first systems level study in grapes leveraged transcriptomic, metabolomic, and proteomic technologies to understand berry development and the postharvest withering process (i.e., controlled dehydration) in cv. “Corvina” grapes (Zamboni et al., 2010). Using a combination of hypothesis-free and -driven integration approaches, the authors were able to tease out putative berry stage-specific functional networks. As an outcome, a fully integrated network related to the withering process revealed key phenylpropanoid and stress-responsive genes (i.e., biotic, osmotic, and oxidative), together with proteins involved in oxidative- and osmotic-stress, and secondary metabolites such as acylated anthocyanins and stilbenes. Recently, integration of berry metabolome (primary and secondary) and proteome networks encompassing 12 developmental stages revealed a greater propensity of an energy-linked metabolism in berries prior to veraison (Wang et al., in press). These observations corroborated earlier studies (Dai et al., 2013; Cuadros-Inostroza et al., 2016), demonstrating that pronounced changes in the berry occurs before veraison, characterized by a reduction of many early accumulating primary metabolites. Interestingly, the integrated network also revealed several modules with high node degree for many metabolites (amino acids and organic acids) and corresponding enzymes catalyzing their synthesis (Wang et al., in press).

Characterizing genes that regulate the accumulation of secondary metabolites throughout fruit ripening is key for improving quality traits and for predicting plant behavior in response to the environment. In this sense, transcript-metabolite associations have been used to prioritize candidate genes important for determining berry quality parameters under adverse environmental conditions (Savoi et al., 2016). Integrated transcript-metabolite networks encompassing monoterpenes that are both ripening-related and drought-modulated (e.g., linalool, nerol, α-terpineol) revealed many highly co-regulated transcripts to be involved in terpene and lipid metabolism. The authors further highlighted VviMYB24 as a promising regulatory candidate for monoterpene biosynthesis given consistent correlations with all three monoterpenes in their study.

*Cis*-regulatory element-driven networks have been recently constructed using integrated information of promoter CRE structure and network connectivity (Wong et al., 2017). Numerous CRE-driven modules inferred using condition-dependent GCNs (development-dynamic and stress-specific) highlighted roles in stress response (e.g., to drought and pathogens) and developmental processes (e.g., fruit ripening). For example, GCC-core sub-modules contained many genes that were highly induced in berries and leaves infected with fungi (Wong et al., 2017).

*Cis*-regulatory element enrichment maps or transcript information for miRNA target enrichment analysis can be easily integrated into plant GCNs. This approach has been used to prioritize target genes of the entire grape R2R3-MYB family (Wong et al., 2016) and also to explain the expression responses of module genes under prolonged drought stress in berries (Savoi et al., 2016). Enrichment for miRNA targets within GCNs has suggested a pivotal role of these molecules in regulating the expression of “switch” genes in a stage-specific manner (Palumbo et al., 2014). Finally, aggregating several networks into a community network can also be advantageous to effectively reveal discrepancies between individual networks while highlighting associations common across individual networks (Proost and Mutwil, 2016). This approach has been used by Loyola et al. (2016) to identify a set of high confidence targets of HY5 and HYH given by the combination of microarray and RNA-Seq data with genome-wide promoter inspections. It is noteworthy that “condition-independent” and “condition-dependent” approaches are still useful for providing a preliminary insight into co-expression relationships in grapes.

## An illustration for the integration of multilayered networks for dissecting the complexity of the berry's phenylpropanoid composition

In grapes, phenylpropanoids influence their organoleptic properties and beneficial attributes to human health, highlighting the importance of their study. Several reports have demonstrated the complex nature of secondary metabolism in grapevine, both at the level of chemical composition and genetic regulation (Dal Santo et al., 2013; Costantini et al., 2015; Malacarne et al., 2015). Among the many phenylpropanoid compounds that influence the quality of grapes and wines, some of the most important are flavonoids (anthocyanins, flavonols and tannins) and stilbenes. These compounds accumulate in a temporal and compartmentalized manner and numerous regulators of their accumulation have been characterized to date (Reviewed by Kuhn et al., 2014; Matus, 2016). One strikingly relevant feature of the grapevine genome is that wine quality-related gene families are expanded in gene number (Martin et al., 2010; Vannozzi et al., 2012), including those related with transcription factor activity (Matus et al., 2008; Wong et al., 2016). Genomics and transcriptomics data originated from these and others studies suggest that the regulation of secondary metabolism in grape is a much more complex trait compared to plant model species. As large-scale omics data are periodically accumulating; there is an enormous potential for gene discovery in relation to grape secondary metabolic pathways.

**Figure 1:**
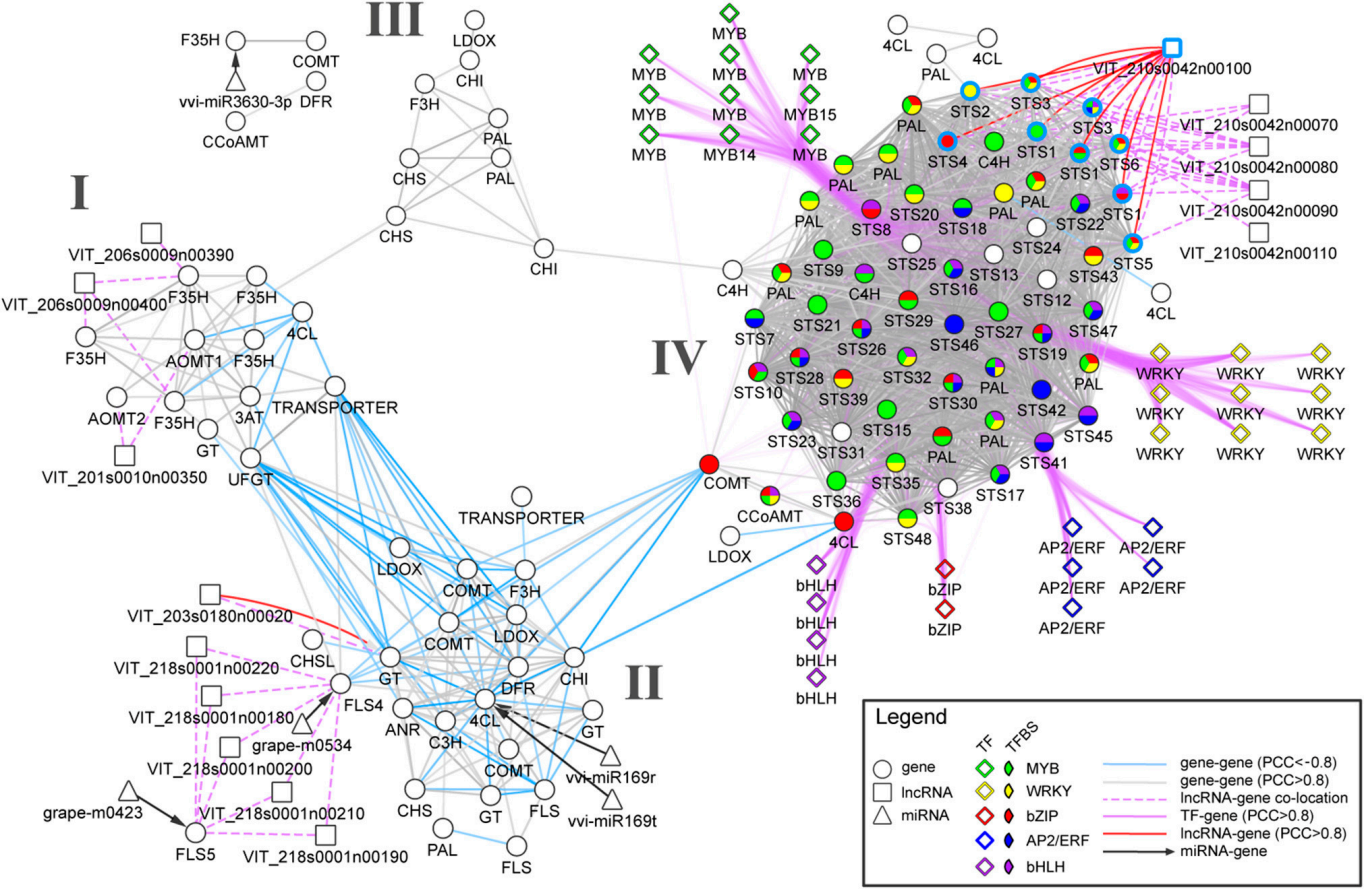
**An integrated network for phenylpropanoid regulation in the grape berry**. Circle, diamond, square, and triangle nodes represent enzyme-coding genes, transcription factors (TFs), long non-coding RNAs (lncRNAs), and micro RNAs (miRNAs), respectively. Gray and light blue solid edges connecting circle and square nodes depict positive and negative RNA-Seq-derived co-expression correlations, respectively. Dashed and dotted lines depict predicted miRNA-gene interaction and lncRNA-gene co-location (within 100 kb). Clusters I, II and III connect genes belonging to the early phenylpropanoid and flavonoid (ePP and Fla) sub-pathways. Cluster IV shows a dense group containing predominantly *PHENYLALANINE AMMONIA-LYASE* (*PAL*) and *STILBENE SYNTHASE* (*STS*) genes that are largely co-expressed with positive co-expression correlations. Purple edges represent positive co-expression correlations between TFs and early phenylpropanoid pathway genes. Pie chart colors represent the presence of selected TF-binding sites (based on *cis*-regulatory element enrichment analysis) in promoter regions of the corresponding enzyme-coding genes. Light blue border edges depict *STS* genes located in chromosome 10.

To demonstrate how various biological networks can be integrated to study berry's phenylpropanoid composition, we gathered networks generated from gene co-expression analyses, predicted miRNA-gene and long non-coding RNA (lncRNA) -gene interactions. First, we re-analyzed a comprehensive berry ripening RNA-Seq transcriptome dataset (five black-skinned cultivars sampled at four developmental stages; Palumbo et al., 2014) and constructed a ripening-specific gene co-expression network (PCC > |0.8|). This ripening-specific GCN was then used as a basis for lncRNA-gene network, which consisted of predicted lncRNAs (Vitulo et al., 2014) that showed strong correlation with a putative “interacting” gene (PCC > | 0.8 |) that was co-located within 100 kb flanking the lncRNA position. Using a comprehensive catalog of grapevine miRNAs (Belli Kullan et al., 2015; Pulvirenti et al., 2015), we also reanalyzed potential miRNA-mRNA interactions using psRNATarget with default parameters (Dai and Zhao, 2011). As the interpretation of each network at a global scale is out of the scope of this perspective, we focused our attention on the early phenylpropanoid and flavonoid (ePP and Fla) pathways and on the potential regulatory genes and their interactions (among genes, miRNAs, and lncRNAs). The resulting network is composed of 112 ePP/Fla pathway genes (differentially expressed during berry development and ripening) together with five miRNA and 14 predicted grapevine lncRNAs (Figure 1). GCN analysis revealed a strong co-regulation within early phenylpropanoid and flavonoid pathway genes maintaining few connections between both sub-pathway genes during the course of berry development and ripening.

Three clusters (I, II and III) were observed for Fla pathway genes sharing many positive correlations within each group (Figure 1). Cluster I contained genes mainly involved in the regulation of anthocyanin accumulation such as five flavonoid-3′,5′-hydroxylases (*F3*′*5*′*H*), two anthocyanin-o-methyltransferases (*AOMT1-2*), the *UDP-GLUCOSE:FLAVONOID 3-O-GLUCOSYLTRANSFERASE* (*UFGT*) and *ANTHOCYANIN-3-O-GLUCOSIDE-6*′′*-O-ACYLTRANSFERASE* (*3AT*). Cluster II consisted of genes encoding proanthocyanidin biosynthesis genes including three predicted galloyl glucosyltransferases, *ANTHOCYANIDIN REDUCTASE* (ANR), and *LEUCOANTHOCYANIDIN REDUCTASE* (LAR), as well as upstream flavonoid pathway genes such as *CHALCONE SYNTHASE* (*CHS*) and *CHALCONE ISOMERASE* (*CHI*). One predicted antisense lncRNA (*VIT_203s0180n00020*) collocated (within 50 kB) and positively correlated with one galloyl glucosyltransferase gene (*VIT_03s0180g00200*). This cluster also contained genes encoding one 4-coumarate-Co-A ligase (4CL), two flavonol synthases (FLS4-5), one flavanone-3-hydroxylase (F3H), and one caffeic acid 3-o-methyltransferase (COMT), all of which were negatively correlated with genes from cluster I. Furthermore, grapevine miRNAs miR169r/t and grape-m0534 were predicted to target *4CL* and *FLS*4, respectively. Several genes belonging to cluster II and I shared negative correlations (light blue solid edges, Figure 1). This separation is evident whereby the majority of genes from cluster I are ripening-specific (i.e., upregulated from veraison onwards), while many genes from cluster II are mostly expressed during the early-to-mid stages of berry development (and subsequently downregulated as ripening progresses).

As there is much less evidence in the regulation of the early phenylpropanoid and stilbene sub-pathways compared to the regulation of flavonoid biosynthesis, we focused our attention on a fourth, highly connected cluster (IV) holding strong positive correlations within and between the two large *PHENYLALANINE AMMONIA-LYASE* (*PAL*) and *STILBENE SYNTHASE* (*STS*) gene families (Figure 1). Two cinnamate-4-hydroxylases (*C4H*) also shared many strong positive correlations with *PAL* genes and one *4CL* was positively correlated with many STS encoding genes. Gene expressions within this cluster were mainly late-ripening specific, with many of them peaking at harvest. Promoters from cluster IV were highly enriched for *cis*-regulatory elements including those for R2R3-MYB, AP2/ERF, WRKY, bHLH, and bZIP TF binding. In particular, the MYB binding site CCWACC was present in one *CCoAMT*, two *C4H*, 10 *PAL*, and 27 *STS* genes. The potential regulation of these genes by MYB transcription factors is supported by recent studies showing that several grapevine MYBs may have regulatory roles controlling the levels of small weight phenylpropanoids and stilbenes (Höll et al., 2013; Cavallini et al., 2015). Our approach is novel in suggesting the regulatory roles by other TF families such as WRKY and AP2/ERF. For example, strong co-regulation of nine WRKY TF to 11 *PAL* and 44 *STS* genes coincided with the presence of WRKY *cis*-regulatory elements in many *PAL* and *STS* genes. Interestingly, one of the four predicted intergenic lncRNAs (*VIT_210s0042n00100*) was co-located and strongly co-regulated with all nine *STS* positioned on chromosome 10. Recent evidence from several functionally characterized lncRNAs in animals and plants suggest that lncRNAs could operate as decoys, guides, signals, and scaffolds, acting as single molecules or complexes regulating pre- and post-transcriptional processes (Wang and Chang, 2011). As such, our observation raises the plausibility of a large-scale regulatory function between this lncRNA and *STS* genes. This *STS*-associated lncRNA may fulfill combinatorial roles for the fine-regulation of multiple *STS*, as signals for transcription activity in a stage-specific way or as guides for chromatin modifiers to the cluster of tandem-positioned *STS* of chromosome 10, potentially modulating DNA accessibility.

## Conclusion

Multi-omics studies incorporating systems biology approaches in grapevine have facilitated the identification of new grape secondary metabolism regulators and have helped in the characterization of genome-wide responses to environmental factors. These studies have brought knowledge and new tools to understand how to modify and improve grape's quality. Additional efforts will still be needed to map protein-DNA and protein-protein landscapes at a large scale. Also, DNAse I hypersensitivity mapping could be useful to identify pioneering transcription factors controlling grape and wine quality traits.

## Author contributions

JTM conceived the article and planned its structure. DW and JTM searched and discussed the literature and wrote the manuscript. DW generated new network data. All authors have read and approved the manuscript.

### Conflict of interest statement

The authors declare that the research was conducted in the absence of any commercial or financial relationships that could be construed as a potential conflict of interest.

## References

[B1] Amato A. Cavallini E. Zenoni S. Finezzo L. Begheldo M. Ruperti B.. (2017). A grapevine TTG2-like WRKY transcription factor is involved in regulating vacuolar transport and flavonoid biosynthesis. Front. Plant Sci. 7:1979. 10.3389/fpls.2016.01979g 28105033 PMC5214514

[B2] Arabidopsis Interactome Mapping Consortium (2011). Evidence for network evolution in an Arabidopsis interactome map. Science 333, 601–607. 10.1126/science.1203877 21798944 PMC3170756

[B3] Belli Kullan J. Lopes Paim Pinto D. Bertolini E. Fasoli M. Zenoni S. Tornielli G. B.. (2015). miRVine: a microRNA expression atlas of grapevine based on small RNA sequencing. BMC Genomics 16:393. 10.1186/s12864-015-1610-5 25981679 PMC4434875

[B4] Cavallini E. Matus J. T. Finezzo L. Zenoni S. Loyola R. Guzzo F.. (2015). The phenylpropanoid pathway is controlled at different branches by a set of R2R3-MYB C2 repressors in grapevine. Plant Physiol. 167, 1448–1470. 10.1104/pp.114.256172 25659381 PMC4378173

[B5] Costantini L. Malacarne G. Lorenzi S. Troggio M. Mattivi F. Moser C.. (2015). New candidate genes for the fine regulation of the colour of grapes. J. Exp. Bot. 66, 4427–4440. 10.1093/jxb/erv159 26071528 PMC4507754

[B6] Cuadros-Inostroza A. Ruíz-Lara S. González E. Eckardt A. Willmitzer L. Peña-Cortés H. (2016). GC-MS metabolic profiling of Cabernet Sauvignon and Merlot cultivars during grapevine berry development and network analysis reveals a stage- and cultivar-dependent connectivity of primary metabolites. Metabolomics 12, 1–17. 10.1007/s11306-015-0927-z 26848290 PMC4723623

[B7] Dai X. Zhao P. X. (2011). PsRNATarget: a plant small RNA target analysis server. Nucleic Acids Res. 39, 155–159. 10.1093/nar/gkr319 21622958 PMC3125753

[B8] Dai Z. W. León C. Feil R. Lunn J. E. Delrot S. Gomès E. (2013). Metabolic profiling reveals coordinated switches in primary carbohydrate metabolism in grape berry (*Vitis vinifera* L.), a non-climacteric fleshy fruit. J. Exp. Bot. 64, 1345–1355. 10.1093/jxb/ers396 23364938 PMC3598422

[B9] Dal Santo S. Tornielli G. B. Zenoni S. Fasoli M. Farina L. Anesi A.. (2013). The plasticity of the grapevine berry transcriptome. Genome Biol. 14:r54. 10.1186/gb-2013-14-6-r54 23759170 PMC3706941

[B10] Degu A. Hochberg U. Sikron N. Venturini L. Buson G. Ghan R.. (2014). Metabolite and transcript profiling of berry skin during fruit development elucidates differential regulation between Cabernet Sauvignon and Shiraz cultivars at branching points in the polyphenol pathway. BMC Plant Biol. 14:188. 10.1186/s12870-014-0188-4 25064275 PMC4222437

[B11] Ferrier T. Matus J. T. Jin J. Riechmann J. L. (2011). Arabidopsis paves the way: genomic and network analyses in crops. Curr. Opin. Biotechnol. 22, 260–270. 10.1016/j.copbio.2010.11.010 21167706

[B12] Gillis J. Pavlidis P. (2012). Guilt by association is the exception rather than the rule in gene networks. PLoS Comput. Biol. 8:e1002444. 10.1371/journal.pcbi.1002444 22479173 PMC3315453

[B13] Guan L. Dai Z. Wu B. H. Wu J. Merlin I. Hilbert G.. (2016). Anthocyanin biosynthesis is differentially regulated by light in the skin and flesh of white-fleshed and teinturier grape berries. Planta 243, 23–41. 10.1007/s00425-015-2391-4 26335854

[B14] Höll J. Vannozzi A. Czemmel S. D'Onofrio C. Walker A. R. Rausch T.. (2013). The R2R3-MYB transcription factors MYB14 and MYB15 regulate stilbene biosynthesis in *Vitis vinifera*. Plant Cell 25, 4135–4149. 10.1105/tpc.113.117127 24151295 PMC3877794

[B15] Itkin M. Heinig U. Tzfadia O. Bhide A. J. Shinde B. Cardenas P. D.. (2013). Biosynthesis of antinutritional alkaloids in solanaceous crops is mediated by clustered genes. Science 341, 175–179. 10.1126/science.1240230 23788733

[B16] Kuhn N. Guan L. Dai Z. W. Wu B. H. Lauvergeat V. Gomès E.. (2014). Berry ripening: recently heard through the grapevine. J. Exp. Bot. 65, 4543–4559. 10.1093/jxb/ert395 24285825

[B17] Loyola R. Herrera D. Mas A. Wong D. C. J. Höll J. Cavallini E.. (2016). The photomorphogenic factors UV-B RECEPTOR 1, ELONGATED HYPOCOTYL 5, and HY5 HOMOLOGUE are part of the UV-B signalling pathway in grapevine and mediate flavonol accumulation in response to the environment. J. Exp. Bot. 67, 5429–5445. 10.1093/jxb/erw307 27543604 PMC5049392

[B18] Malacarne G. Costantini L. Coller E. Battilana J. Velasco R. Vrhovsek U.. (2015). Regulation of flavonol content and composition in (Syrah×Pinot Noir) mature grapes: integration of transcriptional profiling and metabolic quantitative trait locus analyses. J. Exp. Bot. 66, 4441–4453. 10.1093/jxb/erv243 26071529 PMC4507773

[B19] Malacarne G. Coller E. Czemmel S. Vrhovsek U. Engelen K. Goremykin V.. (2016). The grapevine VvibZIPC22 transcription factor is involved in the regulation of flavonoid biosynthesis. J. Exp. Bot. 67, 3509–3522. 10.1093/jxb/erw181 27194742 PMC4892739

[B20] Martin D. M. Aubourg S. Schouwey M. B. Daviet L. Schalk M. Toub O.. (2010). Functional annotation, genome organization and phylogeny of the grapevine (*Vitis vinifera*) terpene synthase gene family based on genome assembly, FLcDNA cloning, and enzyme assays. BMC Plant Biol. 10:226. 10.1186/1471-2229-10-226 20964856 PMC3017849

[B21] Matus J. T. (2016). Transcriptomic and metabolomic setworks in the grape berry illustrate that it takes more than flavonoids to fight against ultraviolet radiation. Front. Plant Sci. 7:1337. 10.3389/fpls.2016.01337 27625679 PMC5003916

[B22] Matus J. T. Aquea F. Arce-Johnson P. (2008). Analysis of the grape MYB R2R3 subfamily reveals expanded wine quality-related clades and conserved gene structure organization across Vitis and Arabidopsis genomes. BMC Plant Biol. 8:83. 10.1186/1471-2229-8-83 18647406 PMC2507771

[B23] Moretto M. Sonego P. Pilati S. Malacarne G. Costantini L. Grzeskowiak L.. (2016). VESPUCCI: exploring patterns of gene expression in grapevine. Front. Plant Sci. 7:633. 10.3389/fpls.2016.00633 27242836 PMC4862315

[B24] Obayashi T. Nishida K. Kasahara K. Kinoshita K. (2011). ATTED-II updates: condition-specific gene coexpression to extend coexpression analyses and applications to a broad range of flowering plants. Plant Cell Physiol. 52, 213–219. 10.1093/pcp/pcq203 21217125 PMC3037081

[B25] Palumbo M. C. Zenoni S. Fasoli M. Massonnet M. Farina L. Castiglione F.. (2014). Integrated network analysis identifies fight-club nodes as a class of hubs encompassing key putative switch genes that induce major transcriptome reprogramming during grapevine development. Plant Cell Online 26, 4617–4635. 10.1105/tpc.114.133710 25490918 PMC4311215

[B26] Persson S. Wei H. Milne J. Page G. P. Somerville C. R. (2005). Identification of genes required for cellulose synthesis by regression analysis of public microarray data sets. Proc. Natl. Acad. Sci. U.S.A. 102, 8633–8638. 10.1073/pnas.0503392102 15932943 PMC1142401

[B27] Proost S. Mutwil M. (2016). Tools of the trade: studying molecular networks in plants. Curr. Opin. Plant Biol. 30, 130–140. 10.1016/j.pbi.2016.02.010 26990519

[B28] Pulvirenti A. Giugno R. Distefano R. Pigola G. Mongiovi M. Giudice G.. (2015). A knowledge base for *Vitis vinifera* functional analysis. BMC Syst. Biol. 9(Suppl. 3):S5. 10.1186/1752-0509-9-S3-S5 26050794 PMC4464603

[B29] Reshef N. Walbaum N. Agam N. Fait A. (2017). Sunlight modulates fruit metabolic profile and shapes the spatial pattern of compound accumulation within the grape cluster. Front. Plant Sci. 8, 1–20. 10.3389/fpls.2017.00070 28203242 PMC5285383

[B30] Savoi S. Wong D. C. Arapitsas P. Miculan M. Bucchetti B. Peterlunger E.. (2016). Transcriptome and metabolite profiling reveals that prolonged drought modulates the phenylpropanoid and terpenoid pathway in white grapes (*Vitis vinifera* L.). BMC Plant Biol. 16:67. 10.1186/s12870-016-0760-1 27001212 PMC4802899

[B31] Usadel B. Obayashi T. Mutwil M. Giorgi F. M. Bassel G. W. Tanimoto M.. (2009). Co-expression tools for plant biology: opportunities for hypothesis generation and caveats. Plant Cell Environ. 32, 1633–1651. 10.1111/j.1365-3040.2009.02040.x 19712066

[B32] Vannozzi A. Dry I. B. Fasoli M. Zenoni S. Lucchin M. (2012). Genome-wide analysis of the grapevine stilbene synthase multigenic family: genomic organization and expression profiles upon biotic and abiotic stresses. BMC Plant Biol. 12:130. 10.1186/1471-2229-12-130 22863370 PMC3433347

[B33] Vitulo N. Forcato C. Carpinelli E. C. Telatin A. Campagna D. D'Angelo M.. (2014). A deep survey of alternative splicing in grape reveals changes in the splicing machinery related to tissue, stress condition and genotype. BMC Plant Biol. 14:99. 10.1186/1471-2229-14-99 24739459 PMC4108029

[B34] Wang K. C. Chang H. Y. (2011). Molecular mechanisms of long noncoding RNAs. Mol. Cell 43, 904–914. 10.1016/j.molcel.2011.08.018 21925379 PMC3199020

[B35] Wang M. Vannozzi A. Wang G. Zhong Y. Corso M. Cavallini E.. (2015). A comprehensive survey of the grapevine VQ gene family and its transcriptional correlation with WRKY proteins. Front. Plant Sci. 6:417. 10.3389/fpls.2015.00417 26124765 PMC4464145

[B36] Wang L. Sun X. Weiszmann J. Weckwerth W. (in press). System-level granger network analysis of integrated proteomic metabolomic dynamics identifies key points of grape berry development at the interface of primary secondary metabolism. Front. Plant Sci. Rev. 10.3389/fpls.2017.01066 PMC5491621 28713396

[B37] Wen Y. Q. Zhong G. Y. Gao Y. Lan Y. B. Duan C. Q. Pan Q. H. (2015). Using the combined analysis of transcripts and metabolites to propose key genes for differential terpene accumulation across two regions. BMC Plant Biol. 15:240. 10.1186/s12870-015-0631-1 26444528 PMC4595271

[B38] Wolfe C. J. Kohane I. S. Butte A. J. (2005). Systematic survey reveals general applicability of guilt-by-association within gene coexpression networks. BMC Bioinformatics 6:227. 10.1186/1471-2105-6-227 16162296 PMC1239911

[B39] Wong D. C. Sweetman C. Drew D. P. Ford C. M. (2013). VTCdb: a gene co-expression database for the crop species *Vitis vinifera* (grapevine). BMC Genomics 14:882. 10.1186/1471-2164-14-882 24341535 PMC3904201

[B40] Wong D. C. J. Schlechter R. Vannozzi A. Höll J. Hmmam I. Bogs J.. (2016). A systems-oriented analysis of the grapevine R2R3-MYB transcription factor family uncovers new insights into the regulation of stilbene accumulation. DNA Res. 23, 451–466. 10.1093/dnares/dsw028 27407139 PMC5066171

[B41] Wong D. C. J. Lopez Gutierrez R. Gambetta G. A. Castellarin S. D. (2017). Genome-wide analysis of cis-regulatory element structure and discovery of motif-driven gene co-expression networks in grapevine. DNA Res. [Epub ahead of print]. 10.1093/dnares/dsw061 28119334 PMC5499852

[B42] Zamboni A. Di Carli M. Guzzo F. Stocchero M. Zenoni S. Ferrarini A.. (2010). Identification of putative stage-specific grapevine berry biomarkers and omics data integration into networks. Plant Physiol. 154, 1439–1459. 10.1104/pp.110.160275 20826702 PMC2971619

[B43] Zhang C. Li G. Wang J. Fang J. (2012). Identification of trans-acting siRNAs and their regulatory cascades in grapevine. Bioinformatics 28, 2561–2568. 10.1093/bioinformatics/bts500 22914222

